# Dr. Stephen W. Williams

**Published:** 1853-10

**Authors:** 


*Dr. Stephen W. Williams.*

We find the following in an Eastern paper, and as we learn that Dr. Williams expects to make his home in future in our own State, we publish it in the Journal, at the same time congratulating the members of the profession in Illinois upon the accession to their ranks of a man so eminently qualified to do honor to our science and to secure for it the respect and confidence of the people. —Eds. Journal.

FRANKLIN CO. DIST. MED. COLLEGE.

The Fellows of this Society held an adjourned meeting at Shelburne Falls, Sept. 7.

The President, Dr. Williams. on taking the chair, announced his intention to withdraw his connection from the Society, in consequence of the contemplated removal of his residence, whereupon the following preamble and resolutions were unanimously passed:—

*Whereas* The Fellows of this Society have heard with regret the determination of Dr. Williams, just expressed, therefore

*Resolved*, That we cordially recommend him to the favorable notice of all kindred bodies, as an exemplary associate; and to the public, as a competent physician and surgeon, well versed in the principles of science and learning and a gentleman of unimpeachable character.

*Resolved*, That this Society express its regard for Dr. Williams by presenting him a gold watch.

*Resolved*, That he be requested to give to this Society a sketch of his professional life.

Agreeably to the second resolution a fine gold watch was presented Dr. Williams by Dr. J. Deane, accompanied by the following remarks:

Sir:—In behalf of the Fellows of this Society I present you this gold watch as an expression of our regard for your character as a gentleman and physician. By your varied attainments in learning and science and by your urbanity and punctilious decorum, you have ever won our confidence and respect, and it cannot but be gratifying to you to know that in all the intimate relations that have so long and so uninterruptedly existed between us, we have never entertained a suspicion of your integrity or your honor. It is therefore with sincere regret on our part that these relations are to be severed, but in going from us, you will unquestionably bear with you our fraternal sympathies and good will. Through the remainder of your useful life, do not doubt that these friends will, while they live, cherish your memory and exult in your prosperity. With these sentiments we offer you this parting gift with the hope that it may measure to you many years of health and happiness and honorable age.

The reply of Dr. Williams was as follows :

Gentlemen :—I can scarcely give utterance to my feelings for the elegant gift of this gold watch as a parting token of your affection ; and for the flattering expressions of your regard for me. Next to the approbation of God and my conscience, is that of my professional brethren, for in the language of Burton, "none but a physician can judge with regard to the qualifications of a medical man."

When I look upon this acceptable present—and it will be my constant occupation—it will not only remind me of the rapid flight of time, but also of your endearing friendship which I can never forget. With most of the Fellows of this Society I have long been on terms of intimacy and I trust we shall part with mutual good will. My warmest thanks are due to you all for the distinguished honors you have conferred upon me and for the confidence with which you have accepted my counsels and advice.

This Society is dear to my heart. For many years I have exerted myself to establish it, and it affords me the highest pleasure to know that I leave it in the keeping of gentlemen who will honor it and themselves by their fidelity to it.

From early life I have been devoted to the profession of medicine and my love for it has been unquenchable ; I early put on the professional armor and have labored unceasingly and I hope not unsuccessfully. I shall yet wear it (and perchance die in it) on the lovely prairies of the West, and I shall look back upon your friendship with unmingled satisfaction and delight. In tearing myself away from my beautiful native town where I have resided more than sixty years, I feel that the ligaments of my heart are broken ; but the calls of duty urge me, and they are imperious.

In accordance with the last resolution, Dr. Williams gave a highly interesting account of his professional career, a copy of which was solicited for the archives of the Society.

The usual address was delivered by Dr. Cooke, of Wendell, which was received with zest and instruction and with the thanks of the Fellows. His subject was *Medical Delusions*.

The meeting was closed by a discussion on Dysentery, in which most of the Fellows participated, and was followed by a very excellent dinner served with taste and attention by the gentlemanly proprietor of the Shelburne Falls House. After requesting the editors of the Boston Medical and Surgical Journal and of the Greenfield papers, to publish these proceedings, the Fellows separated, delighted with their interview, not forgetting, however, that it was the last at which the venerable President would preside.

The watch was a heavy full jewelled English lever, of the value of $100, and was purchased of Mr. Josiah Day. It bore upon its outside the following inscription :

STEPHEN WEST WILLIAMS, M. D.
President
Franklin Dist. Med. Society.
From the Fellows,
Sept. 7,
1853.

